# The Limitations of the Healing Art

**Published:** 1890-07

**Authors:** D. R. Wallace

**Affiliations:** Superintendent State Lunatic Asylum, Terrell, Texas


DANIEL'S

### Texas Medical Journal.

A Representative Organ of the Medical Profession, and an Exponent of Rational Medicine; devoted to the Organization, Advancement and Elevation of the Profession in Texas.

PUBLISHED MONTHLY.—SUBSCRIPTION $2.00 A YEAR.

Vol. VI. AUSTIN, JULY, 1890. No. 1.

### Original Articles.

CONTRIBUTED EXCLUSIVELY TO THIS JOURNAL.

*The Articles in this Department are accepted and published with the understanding that we are not responsible for, nor do we indorse the views and opinions of the writers by so doing.*

---

For Daniel's Texas Medical Journal.

### THE LIMITATIONS OF THE HEALING ART.

---

**Odds and Ends of Medical Reading and Observation.**

---

BY D. R. WALLACE, M. D., LL. D., SUPERINTENDENT STATE LUNATIC ASYLUM, TERRELL, TEXAS.

---

[Read before the Terrell Medical Society July 8, 1890.]

IN AN old book, written by one born sixty-five years before the era of grace, familiar to my school years—a book over which many a school boy has sweated, and of which men know too little, may be found these words of advice to writers:

**Sumite materiam uestris, qui scribitis æquam*
*Viribus et versate dia quid ferre recusent*
*Quid valent hameri.*"

Words that might be made to do service at the head of this paper in place of those selected; and at the same time furnish a most instructive and excellent motto to members of the healing art. Literally translated they read—"Oh ye who write, select subject matter suited to your ability, and revolve long what your powers may decline and to what prove equal." A more liberal translation, one making it applicable to the Doctor rather than the scribbler might read,—"Oh ye who practice the healing art would you do straight work and an honest job, would you render a *quid pro quo*, give your clients the worth of their money"; and would you sink into a quiet, peaceable slumber at the end of your day's work, untroubled by qualms of conscience and uneasy forebodings as to how your patients will be on to-morrow, select your cases with care, and revolve long which ones you can safely tackle with your pills, powders and potions, and which, it were better for the patients, as well as your own peace of mind and reputation to leave to the *vis medicatrix naturæ*, diet, nursing and hygienic surroundings; otherwise, it is to be feared that your practice will be what Voltaire says the medical practice was in his time, viz: "the pouring of medicines, of which the Doctor knows little, into stomachs of which he knows less, for diseases about which he knows nothing."

Man and brother, did you never ride slowly homeward from the house of mourning, with a heavy heart, communing with your inner self, clashing fact, against fact by the light of which, questions such as these, in spite of yourself, would take shape in your mind;—ought not this patient to have gotten well? Why did he die? Did my treatment have nothing to do with it—am I sure? Did I give nothing for which there was not clear unmistakable indication? Did I avail myself of all the resources of the healing art, nor go beyond them? Have I the natural aptitude for this high calling, involving the issue of life and death?

*38, 39 and 40. 2 Horatii Flacci Ars Poetica.

Have I the intellectual training and discipline needed to understand the complicated problems of disease? Have I availed myself of all the information within reach? You never have? Then you are more or less than a man.

I knew a man, knew him well, who during the first years of his professional life—I suspect he has long since become hardened —was so set about and sorely pressed by such questionings that though fortified by three diplomas, all bearing on their face *pre-man gradum*, one academic or scholastic, the other two medical, he would have gladly escaped out of their reach, if he had had any place to escape to, where he could have earned his bread by the most servile labor, and eaten it in that obscurity he often felt was his place by nature.

I knew another, one of nature's noblemen and one of the most accomplished medical men of his age I ever knew, who after having spent years in preliminary and professional studies, abandoned the profession in less than three months after he had entered actively upon it; nor could any remonstrance or persuasion on the part of his friends induce him to resume it. His answer was—I am unequal to its responsibilities.

I have known another man, perhaps I have known more than one, who with the preliminary education of a hod-carrier, and with the densest ignorance of medicine, would affect to look down with a lordly contempt upon unassuming men who had forgotten more asleep than he was capable of knowing.

We have all felt pressed to the earth by the weight of responsibility attaching to us, as in some sort held to answer for the health and life of our fellow mortals to the extent our calling may justly be so regarded; when I say all, of course I do not refer to those human sharks that depredate upon society for a living, in the name of the medical art, uncaring and unconcerned about what becomes of their victim; who doubtless feel the better for the knowledge that they get something for nothing. If such creatures did not dead-beat their way through life, as so-called Doctors, they would be dead-beats all the same. Not so all—far from it. I have known many to whom the skeleton in their closets, the bete noir of their lives, was the fear that their professional services were not worth the money they received for them.

Our times constitute an epoch. There have been many like, but none equal to it. Intellectual awakenings resulting in mental activities and material development constitute these epochs in the world's progress. We honor Hippocrates as the father of medicine. He lived in the age of Pericles—the glory of Athens, and one of the greatest statesman of antiquity; of Herodotus, the father of history; of Thucydides, the chronicler of the Peloponesian war, a historic production never equalled or likely to be, standing as it has done, and will continue to, at once the model and despair of all future historians; of Xenophon, only less as an historian than the immortal Thucydides; of Socrates, the greatest, purest and wisest of men, who is credited with having brought philosophy from heaven to dwell among mortals; of Plato and Aristotle, of whom our great Harvard scholar and late Minister to the Court of St. James, says: "He who has read Aristotle will be apt to think that on most points of general applicability, observation has said its last word, and he who has mounted the tower of Plato will never hope to climb another with so lofty a vantage of speculation;" of Democrites, the laughing, and Heraclitus, the weeping philosopher,—the one the father of the automistic theory of the world, the other entitled to the thanks of mankind for the noble sentiment that moral infirmity ought to be pitied, not punished, as a man who should tear out his own eyes would deserve more pity than an ordinary blind man, a sentiment that found utterance, in later times, in the prayer of the Mohammedan philosopher—"Oh God, be kind to the wicked, to the good thou hast been sufficiently so, in that thou hast made them good;" of Euripides, whose tragic splendor has never been equaled; of Sophocles, of Æschylus, of Aristophanes—but time fails to mention more. But these names, familiar to all historians, justify the statement that the age of Pericles was one of an intellectual activity unrivaled in the annals of mankind. Is it a matter of wonder that just at such a time the father of medicine should appear upon the stage? Of the next greatest name that adorns the medical annals of antiquity it is enough to say, Galen was the physician of Marcus Aurelius, and a product of the Augustine age of Latin literature, as Hypocrites was of the Periclean age of Grecian literature. Of the intellectual awakening consequent upon Alexander's expedition into the East and the resulting establishment of the great schools of Alexandria—the wonder and glory of antiquity, let Herophilas who is accused by Tertullian of having made six hundred dissections of the human body, and Erasistratus, who introduced his mild antiphlogistic treatment in place of the polypharmacy and antidotal practice of his time, stand as the medical representatives. Of that wonderful period of intellectual activity, stirred into life by the career of Mohammed and his successors, which aroused Europe from the sleep of centuries of superstition, and left its impress ineffacably graven upon the world's history, as evidenced by such Arabic words as sirup, julip, elixir, admiral, alchymy, alcohol, algebra, chemise, cotton, and hundreds of other words found to-day in English dictionaries, the student of American annals will recall the names of Avicenna, Avenzour, Rhages, Albucasis, and last, not least, the great Alhazan, greater as a philosopher and astronomer than a physician. Humboldt tells us that the Arabs ruled the Christian schools by the study of medicine, to which they added the department of medical pharmacy.

It will be recollected that Vesalius was a cotemporary of Luther and court physician of Charles V., that Harvey was the product of the Elizabethan age of English literature, and that the great Bacon was at once his teacher and his patient. "Is it to be looked at as a mere accidental coincidence that while Napoleon was modernizing the political world, Bichat was revolutionizing the science of life, and the art that is founded on it,—that while the young General was scaling the Alps, the young Surgeon was climbing the steepest summit of unexplored nature,"—that the same year read the announcement of those admirable "researches on life and death" and "the bulletin of the battle of Marengo."

This coup d'oeil is no foolish attempt to display learning; I have none to display. Its introduction is for a purpose. Is there seen no parallelism between these great epochs in the world's advance, and the age in which we live—in this century of steam and electrical dynamics—of railroads and ocean steamers, of telegraphs and telephones, of a Watts, an Arkwright, Compton, Howe, Morse, and Edison, of a Humboldt, Thompson,

Darwin, Tyndall, Huxley, Farraday, and a Herbert Spencer? Is it probable that amid the stir, whirl and shaking up of ideas in every other department of human thought, the medical would not share in the general movement? Surely, no. The medical sciences have advanced more in the last fifty years than in the previous thousand. And for this very reason there has been no time in its history when medical men should be more alive to their resposibility to their fellow-men. *Noblesse oblige.* Great use and great abuse go together. No unmixed good among mortals. Medical practice is an art; nor is any large portion of it based upon exact science. It is largely empirical, and must be so for a long time yet. Here is offered an inviting field for the charlatan to ply his trade. Never was there such urgent demand for the conscientious scientific medical man. Nor is it the outside quack only that needs attention. There is quite as much quackery in the profession, perhaps more, than outside, with a piece of parchment they call a diploma, empowering them to depredate to their heart's content upon the sick and afflicted. This is the worst species of quackery. Being legalized, it is more difficult to reach. It is protected by law and public, even medical opinion. This abuse has become so rank and monstrous, as to make prudent medical men question, as did the Abernathys, Sydenhams and Sir Astley Coopers in their day, whether, medicine taken as a whole—much good as it is confessedly doing—is not doing more harm than good. This, however, is no argument against legitimate medicine. It is an excuse for, if it does not justify the language in a recent issue of the Popular Monthly Science. The writer says: "The time was when it was supposed that the State had to look after the spiritual health of individuals; and for that purpose to prescribe their theological belief and religious observances. That belief has for the most part been exploded in the modern world, but its place has been taken by the notion that the State is responsible for the intellectual health of its members; and in lieu of the State church we have the State schools. As regards the physical health of the community, the general method is to legalize one or two---possibly quite conflicting---schools of medicine, and to empower them to rule out, and, if necessary, to prosecute and punish all others.

"Nobody, broadly speaking, seems to believe that in the absence of all legislation of this character, people could in any adequate manner preserve their health, or protect themselves against gross imposture. We believe it---believe it most heartily; and we believe that the science of medicine would advance far more rapidly, and that on the whole, the public health would be far better, if every man were left perfectly free to employ any one to attend him in sickness. At present every licensed practitioner feels himself authorized to call every unlicensed practitioner a quack. We should prefer a system under which to a quickened public intelligence in questions of health and disease, the quack should stand revealed by his quackery. How much of real quackery is now concealed by the license to practice, it might distress a confiding public to know."

It is proposed to occupy the rest of the time, so kindly accorded me, with a more direct examination of our subject---the limitations of the healing art. It will not fail to occur to you that our art is no exception. Turn wherever he may, man finds himself in all the relations of life and thought, hedged about and closed in by inexorable limitations of his faculties and boundaries of his possible knowledge! While there is a *ne plus ultra* everywhere, the boundaries of knowledge lie in an undiscovered region—no man having yet reached it, however busy in his hunt for it—not even the Plinys, Humboldts and Darwins; while the powers of the mind are susceptible of almost infinite growth in sight and expansion.

> "He that doth the best his circumstances allow,
> Does well—acts nobly; angels can do no more,"—

should be at once the guide and inspiration of every medical man who undertakes the responsible office of looking after the health and lives of his fellow mortals.

I knew, in early life, a very eminent physician who told me that when he came to the practice he was the owner of three medical books; that when he went forth to combat disease, his chief armamentarium consisted of notes taken in the lecture room of the University of Pennsylvania. He was a pupil of Rush, Physic and Chapman. Then there was much reliance upon traditional knowledge, consisting in large part of the experience of the teacher; now, like all other departments of human thought and activity, the medical sciences and the art based on them are embodied in their own appropriate literature.

DOCTORS MUST READ.

What must they read? You have all read the answer of the great Sydenham when Sir Richard Blackmore asked him what medical books he should read. "Read Don Quixote," replied the greatest physician of the seventeenth century. Would one not be about as profitably employed now in reading the great Cervantes' story of the redoubtable Knight of Salamanca and his faithful squire, Sancho Panza, as in reading much that passes as medical literature?

Thousands of new so-called medicinal agents are struggling for recognition. The press is turning off volumes of medical literature by the thousand. The medical journals, "all useful, we hope, all necessary, we trust, many of them excellently well conducted, but which must find something to fill their columns, and so print all the new plans of treatment and new remedies," pile up our study tables. It is matter of wonder—no philosopher having explained it as yet, I believe—what a number of doctors are laboring under a species of *lead poisoning*. A great medical humorist assures us that it is within his own experience—for he contracted the disease early in life—there is no poison so subtle, so diffusive into the whole mass of the blood, as lead poison, contracted from type metal. Once it has reached the cerebral convolutions, the result is a peculiar disease known to the nosologist of the compositors' room as cacoethes scribendi; a disease that is inexpungable, irremediable; not all the hellebore of three corcyras, I am assured, can purge it away. The evidences of the ravages of this fell malady fill our medical journals to overflowing.

If Apollo comes not to the help of us belabored medical mortals, it is not easy to foretell what will become of us. Already the mass of new remedies, whose potencies are unbounded over all the aches and ills flesh has become afflicted with from the eating of an apple, is portentous indeed, and, it seems likely enough, from the present rate of supply, that they will, in the no distant future, be quite sufficient to fill a Chinese encyclopædia.

Man and brother possess your spirit in peace and serenity. Do not permit yourself to be disquieted or to lose sleep. Ninety-nine out of every hundred of these thousand and one drugs, vaunted as world wonders, if not as actual specifics, are entirely inert; and in most of the few exceptionable cases of those which have some virtue of the sort claimed, there are well known established agents, whose remedial powers are familiar, far more efficacious. You can count on your fingers all the really valuable additions to the pharmacopœia made in a single lifetime. With the uses and properties of these you will not have to sit up late at nights to become acquainted. But young America must have room to expand in medicine as in everything else. Says the laughing Doctor Oliver Wendell Holmes:

"How could a people that have a revolution every four years, that have contrived the bowie-knife and six-shooter, that have chewed the juice out of all the superlatives of the language in fourth of July orations, that have so used up its ephithets of abuse that it takes two quarto dictionaries to supply the demand; that insist in sending forth yachts, horses and boys to outrun, out-fight, outsail and checkmate all the rest of creation; how could such a people be content with less than an heroic practice," and he might have added: with a hundred new remedies each year to impart novelty to the effect.

"What wonder," he continues, "that the Stars and Stripes wave over doses of ninety grains of quinine, and that the American eagle screams with delight to see three drachms of calomel go down in a single mouthful."

What becomes, one is tempted to ask, of the hundreds of specifics, with the laudations of whose miracle cures our medical monthlies—no, monthlies are too slow, it requires weeklies—are filled? Specifics indeed! Yes when we have specifically diseased specific men and women to re-treat. Yes, there may be specifics when we can localize the tide and provincialize the law of gravitation; in the words of another: "When an oil is discovered that will make a bad watch keep good time, and when a prescription is found that will turn an acephalous fœtus into a promising child, and a man can enter a second time into his mother's womb and give back to her the infirmities that twenty generations have stirred into her blood and infused into his own through her's, we may have specifics for everything except old age, and perhaps for that too.''

There would seem to be in medical minds of a certain type, an attraction for odd fancies and paralogisms which lead them to juggle with nature—to delve in bogs and fens, infested with will-o-the-wisps, as naturally as chlorotic girls eat chalk, slate-pencils and charcoal. They never seem quite happy unless they are pouring drugs down the throat of some luckless wight, and the newer, and the less they know of them the more scientific they seem to regard their practice.

I am not disposed to cry down new remedies. Not I. They are too few and precious. I saw a statement, about twenty-five years ago, to the effect that the average life of the doctor in active practice, is only about seven years. Mine, according to this statement, has been protracted to over five times the average duration. Would yon like to know how I feel in my professional old age? Well, then I must tell you frankly: I by no means feel alarmed by any very impending danger of being overwhelmed in my old age by new remedies.

The question recurs: What shall we read? Everything? Better read nothing. Indulge me to say this, the medical man possessing that insight born of a disciplined intellect, guided by well grounded knowledge of the medical sciences, knows as instinctively and unerringly what to read as soon as he runs his eyes over the lines, as the Jew dry-goods merchant knows the value of the textile fabric when he creases it back and forth between his fingers and thumb. He is in no more danger of reading a worthless book or worthless article in a journal, than he is of making a full meal from a joint of spoiled meat.

A medical man, whose fame is in no wise confined to the Western Hemisphere, and withal a gentleman of great prudence and temperance of speech, holds this language:

''The journals contain much that is crude and unsound. The presumption is against novelties, unless they come from observers of established credit, and yet I have known a practitioner—perhaps more than one—who was as much under the influence of the last article he read in his medical journal, as the milliner is under the influence of the last fashion plates."

New remedies ! Are not the journals full of the curative wonders of these new remedies? They give the facts, and the facts are facts. Certainly they are, and out-lie everything except figures. You, who talk about facts and figures in medicine, let me stir up your pure minds by way of remembrance. Remember the "Royal Touch" for scrofula; the hundreds of thousands of cures of one of the most intractible of maladies, a remedy sovereign in fact as in name, from the time of Edward the Confessor in 1042, to the accession of Queen Anne, of blessed memory, in 1702, curing all alike: priest, peasant, prince and philosopher, for twenty generations. Remember Unguentum Armarium, weapon ointment, which healed thousands of wounds all over Europe by being mearly applied to the weapons causing them. Remember the Sympathetic Powders, the secret of which could only be purchased for immense sums; their virtues are attested by great offices of the church and state, even by pope and and king. Disolved in a little water, and the solution applied to the blood-stained garment of the wounded person, even though at a great distance, the pain ceased at once, the cure was assured. What was this powder? Simply sulphate of zinc and nothing more. Remember the Metalic Tractors, of the redoubtable Elisha Perkins, which facts and figures show, cured a million and a half of persons in a few months. For about fifteen years, say from 1797 to 1811, they were the cure of all the world, being introduced into Royal Hospitals and Perkinian Institutes all over Europe, Denmark and England leading the way. What were these wonderful tractors, whose mere touch relieved pain and cured disease as if by magic, as attested by great preachers, professors and statesmen? Do not be frightened, you need not hold your breath. Little pieces of metal about three inches long, one iron and the other brass, sharp at one end. Nurses could apply them, and no disease could resist their power. What has become of them? They have not been seen or heard of in a sick room for nearly three-quarters of a century, and it is questionable whether a curiosity hunter would be able, for love or money, to add them to his collection of forgotten things. Thousands of sets were sold for twenty-five dollars apiece, and history tells us that Doctor Perkins' son realized ten thousand pounds sterling by their sale in England alone in three years. Good things, Perkins' Tractors! Shall I say remember the amiable Bishop Berkeley's Tar-water, which cured, in the Bishop's account of it, small-pox, consumption, pleurisy, pneumonia, asthma, dyspepsia, gout and fevers of all kind, besides being a great blood purifier? Shall I come to a close by saying: Remember the greatest *cure all* of modern times, whose whole materia medica consists, as you all know of nothing in the world but sugar of milk, and nomenclature?

Oh, ye who talk of facts and figures in medicine, how do these look? Here are facts and figures for your edification—facts and figures, lies, "the stuff that dreams are made on." Of course, I mean not the facts and figures of prudent, painstaking medical men of credit, but those from nameless sources, arrayed in weeklies and monthlies—written most generally by persons laboring under typical *lead poisoning*, and who have it bad, too bad for the facts and figures to be of any good.

Ninety-three years ago the venerable Dr. James Jackson, for nearly fourty years Professor of Practice in Harvard University, went to Dr. Holyoke, of Salem. "You see," says Dr. Holyoke, addressing his pupil, and pointing to the shelves in his shop, "there seems a great variety of medine here, and that it will take you a long time to learn them all; but most of them are unimportant. These four: mercury, barks, antimony and opium, worth all the rest." Brother, in the healing art, how many have you "worth all the rest?"

I submit, is it not the part of common sense, safest for all concerned, to trust to the old reliables with whose properties you are familiar, rather than to exploit with new remedies whose therapeutical virtues, conceding them to have any, are very imperfectly known to you or others?

What proportion of cases, treated actively with drugs, would be better without medicinal interference, we have no means of knowing; and, I am unwilling to risk your good opinion by hazarding a conjecture. I may be permitted to say, I trust, without offence to my medical brethern, it is the result of nearly forty years observation, that great harm is done, much pain caused, disease aggravated and protracted, even lives jeopardized, by poly-pharmacy, or needless drugging. To drug for every ache and ill I know is expected, particularly by the more ignorant.

The temptation to do what one knows is expected, is great, how great, most of you know. The Sydenhams, Abernethys, Hunters, Sir Astley Coopers, Jacksons and Flints, can afford, you say, perhaps, to put upon diet, order horse-back exercise, hygenic surroundings, and leave the rest to nature; but if we do, our patrons will leave us to starve. Well then, perhaps we ought to starve. Certainly we ought to, if we only keep ourselves alive by doing what we know to be wrong. (!) In those cases in which you know you are expected to give medicine where none is needed, and you have not the "back-bone" to say so, would it not be better for your patient as well as for your reputation to give bread pills, starch powders, tinctures of exonphalos or fluid extract of abracadabra?

Because not drugging him, follows it that the doctor is doing nothing for his patient? In a letter to a young physician, Dr. Jackson, of Harvard, already alluded to, writes:

"It is a very narrow and unjust view of medicine, to suppose it consists in the use of powerful drugs, or of drugs of any kind. The doctor can do much for the sick, more than others can, though in the major part of the cases he does not undertake to control or overcome the disease by art." This great and conscientious medical man tells us in the same letter, that he never reports cases *cured* in the hospital. When recovered he reported them *well.*

Perhaps in the no distant future—obtaining a knowledge of the medical science, and acquiring the art of healing, will be entirely distinct;---the former being taught in the colleges and universities, and the latter in the wards of hospitals by clinical lectures ---a diploma of proficiency from the schools of science being an indispensable pre-requisite to enter upon the study of the healing art at all. This was the view entertained by the great Jefferson, who, sending his nephew and ward to Philadelphia to study the medical science, wrote to Dr. Physic that he did not desire him to study practice, midwifery, and surgery. "In Medicine of the Future," an address prepared for the British Medical Association—he died before the Association met—the late Austin Flint gives it as his opinion that the teaching of the healing art, its practice and technique, will be in the near future almost, if not wholly, clinical. Of the practice he says: "It is a pleasant thought that hereafter the practice of medicine may not be so closely interwoven in the popular mind with the use of drugs. The time will come, when the visit of the physician will not, as a matter of course, involve the co-operation of the pharmacist. When this time comes a system of practice which assumes to substitute medicinal dynamics for the *vis medicatrix naturæ* will have been added to the list of by-gone medical delusions."

The medical sciences belong to the department of positive knowledge, and should be taught as such; but, the art of healing is now, and must for long continue to be, largely empirical. A glance at our very imperfect idea of the modus operandi of medicinal agents, in every day use, suffices to prove this, without taking into the account the numberless exemplification of it, with which medical history abounds. You do not need for me to tell you that the healing art learned the use of calomel from a charlatan; of antimony from a monk; to cure ague from a Jesuit; lithotomy from a friar; to treat gout from a soldier; to keep off scurvy from a sailor; from a clerk to sound the eustachian tube; to cure itch from a wash-woman; from a milk-maid to guard against small-pox; from a buccaneer the use of the popular compound, opium; acupuncture and the moxa from the Asiatic heathen; the use of lobelia from the American savages; and, last but not least to trust to, not try to supplant, the resources of nature, from Hahnemann and his followers.

But having detained you long enough, I round to a finish, with words, which, while precisely expressing the thought I desire to leave with you on parting, are so much prettier and expressive than any that would be likely to occur to my dull fancy, I know you will pardon my appropriating them. They are these:

"Friends and brothers, there is nothing to be feared from any seeming heresy to which you may have listened. I cannot compromise your collective wisdom. If I have strained the truth one hair's breadth for the sake of an epigram or an antithesis, you are accustomed to count the normal pulse-beats of sound judgment, and know full well how to recognize the fever-throbs of conceit and the nervous palpitations of rhetoric!"

